# Relationships between Dota 2 expertise and decision-making ability

**DOI:** 10.1371/journal.pone.0264350

**Published:** 2022-03-01

**Authors:** Daniel Eriksson Sörman, Karl Eriksson Dahl, Daniel Lindmark, Patrik Hansson, Mariana Vega-Mendoza, Jessica Körning-Ljungberg

**Affiliations:** 1 Department of Health, Education, and Technology, Luleå University of Technology, Luleå, Sweden; 2 Department of Psychology, Umeå University, Umeå, Sweden; Texas A&M University, UNITED STATES

## Abstract

Esports is an often time-consuming activity that has become increasingly popular with billions of players all over the world. The objective of this study was to investigate if there is a relationship between skill level in the strategy video game Dota 2, a game that places many demands on decision making to be successful, and decision making under ambiguity and experience as measured by performance in the Iowa Gambling Task (IGT), a task known to have ecological validity. Two indicators of players’ performance in Dota 2, namely match-making rating (MMR) and Medal, were used as predictors of performance in the IGT in path models. Results showed that Medal was a significant predictor of performance in IGT, while MMR score was borderline significant. The cognitive reflection task, included in the models as an indicator of the ability to engage in conscious, analytical, rational, and logical thinking, was a significant predictor of performance in IGT, and was significantly and positively related to MMR. The findings from this study give insight into the cognitive demands related to performance in Dota 2. Although results suggest that strategy video gaming may be a factor that contributes to increased decision making abilities, a reversed relationship is also possible, whereby individuals who are better at decision making are also more likely to become successful in Dota-2. More studies, preferably longitudinal, are needed to replicate the findings of this study and to establish the directionality between factors.

## Introduction

The use of video games for entertainment has become a worldwide phenomenon, and it is estimated that there are over 2.5 billion video game players across the world [1]. The growing popularity of video games over the last decades has increased scholars’ interest in examining different behavioral and cognitive aspects related to gaming (see e.g., [2–4]).

An important issue concerns whether video gaming can be used as a tool for learning-induced plasticity [5]. The interest in “brain training” has often focused on, but has not been restricted to, research on the elderly and has resulted in a surge of computerized cognitive interventions. Beneficial effects of cognitive training have been found in different cognitive domains, such as working memory [6,7], attention [8] and inhibitory control [9]. Despite this, however, the efficacy of computerized training is controversial and has been criticized for its lack of generalizability (i.e. transfer) to tasks other than those specifically trained [10,11]. Nonetheless, the possibility for computerized cognitive training has posed the question whether cognitive training through video gaming can produce transfer effects to other areas, and whether video gaming can be used as a form of neurocognitive training in different age groups (see e.g., [12–16]).

However, as for cognitive training as a whole, there is no agreement as to whether video gaming can produce cognitive benefits. Some meta-analyses (e.g., [16]) suggest that videogames can improve cognitive functioning, such as reaction time, attention, and memory, while some report minor to non-effects [17]. Nonetheless, even if it is possible that extensive experience with video gaming has the potential to produce beneficial effects on tasks other than those specifically trained, the question remains if transfer can be found on more general cognitive tasks (far transfer), or if it is restricted to closely related tasks (near transfer) [18].

In addition, even if it is possible that long-term exposure to video gaming can promote cognitive functions, it must be stressed that so far, most studies in this area are cross-sectional, and thus the directionality between gaming experience and cognitive performance is hard to establish. Thus, it is plausible that high cognitive ability can be considered as a factor that increases the likelihood of becoming successful in video games. Such possible directionality between factors may be of interest for the gaming society. Today, e-sports are becoming increasingly professionally oriented. Consequently, results from cognitive tests can potentially be used as a screening tool with the purpose of identifying plausible future elite players. Large et al [19] emphasizes that, just as cognitive abilities can be used as a selection criterion in other branches, further understanding of the cognitive underpinnings in e-sports may increase the likelihood of recruiting individuals more likely of success in video games.

Regardless of directionality, results from correlational studies can explain some of the cognitive demands associated with video gaming. More knowledge of how cognitive demands can be directly tied to in-game challenges and difficulties may also increase the understanding of how video game performance may be associated with different behavioral outcomes [20].

Most of the research in this area has been conducted on the genre of action video games where the most stable, although not undisputed, effects have been found on perception, visual attention and top-down attention [21–23], but also on working memory capacity, speed of processing, and deductive reasoning (see e.g., [20]). Studies on more complex cognitive functions have been inconclusive, with for instance Bailey, West and Kuffel [24] showing associations between video game play and disadvantageous decision-making, while Buelow, Okdie and Cooper [25] found that exposure to video games was associated with faster learning on a decision-making task.

Related to this, it is important to note that there is a wide range of experiences available under the category of ‘video games’, and that the label includes experiences which to a large extent differ from each other. As Bediou et al. [21] puts it; “a superordinate category label such as video games is likely to have limited predictive power” (p. 79) when different games pose different demands to our cognitive, attentional and perceptual abilities. Many studies have tried to classify video games to fall under different game genres (see e.g., [23]). But classification of games is not always a straightforward task, and this has to some extent limited the generalizability and conclusions of the research findings. Similarly, the literature is diverse regarding how to categorize the so-called ‘Multiplayer online battle arena’ (MOBA) games. Games that fall under MOBA are in turn sometimes referred to as ‘action real-time strategy games’ (ARTS; see e.g., [26–28]), which is supposed to underscore that they have elements of both ‘action video games’ (AVG) and ‘real-time strategy’ (RTS) games. However, MOBAs have sometimes been folded into either AVG or RTS categories (see e.g., [29–31]. The question is whether it is even possible to fully establish a genre for specific games, and even more so from a long-term perspective. As highlighted by Dale et al [32], video gaming is an ever-changing landscape, and it is therefore important that researchers are familiar with how games continuously change.

With the rise of popularity of MOBAs, the interest of studying such games has increased [33]. Because of the difference in pace and strategizing in MOBAs, there are reasons to believe that different demands are put on the player. Games within the MOBA genre entail a large number of variables affecting the game state. The player is required to make decisions under real life circumstances where the outcomes or their probabilities are not entirely known, a situation known in behavioral economics as *decision-making under ambiguity* [34]. Among these games is Dota 2, widely regarded as being one of the most complex with regards to interplay between game elements.

Dota 2 is a free-to-play MOBA played on personal computers (PC). Concurrent Dota 2 players during 2021 averaged around 500 000 [35], and with approximately 7.6 million monthly active users worldwide [36]. Dota 2 is known for having one of the highest accumulated prize pools in esports, which is distributed among the professional players [37]. A game consists of two teams of five players each with the end goal of destroying the enemy base. Before the game begins, players can choose from 117 playable characters. Each playable character has a set of at least 4 distinct abilities. Together with 224 different items, some with their own abilities available throughout the game, a large amount of complex interactions is made available for the player which can influence the game state. Knowledge of the vast number of distinct game play elements, their interactions and the ability to predict them, are crucial to success.

To date, some studies have explored relationships between cognitive domains and MOBA performance, most commonly among League of Legends (LoL) players. Kokkinakis et al [38] found a positive but moderate correlation between game expertise (i.e., ranking in LoL) and fluid intelligence, as measured by a WASI-II Matrix Subset [39] in a sample of young adults. Large et al [19] found expertise in LoL to be positively associated with speed of processing and attentional control in a sample of participants aged 18 to 56 years. Other studies have investigated relationships between LoL and cognitive performance among university students; Yao et al [28] found that experts in LoL had larger visual working memory capacity compared to non-experts. Gan et al [26] found that experts in LoL showed superior temporal visual selective attention compared to non-experts, and had also faster information processing. Similarly, Qui et al [31] found that playing LoL improved response times in visual selective attention, which was evident both among experts and non-experts. Finally, Ding et al. [40] investigated behavioral correlates related to MOBA, and found a modest correlation between ranking in LoL and conscientiousness.

MOBA players have also been included in other studies. However, some of these (e.g., [29,30]) have, as noted above, folded MOBA games into a game genre (e.g., RTS), which has made it difficult to establish relationships between specific MOBAs and cognitive performance. Dale and Green [30], for instance, acknowledged that games that fall into the MOBA category contain more action components than regular RTS games, and also suggested that future studies rather should move away from genre-based classifications and instead focus on the actual mechanics of the games that are played.

With regards to Dota 2, a study on game expertise and attentional allocation [41] showed that experts in Dota 2 had higher transition patterns compared to beginners in an in-game eye-tracking test, meaning that highly skilled players could make more use of their cognitive capability. Others have explored whether personality and working memory capacity predict performance (Match-making rating) in Dota 2 [42], and reported that personality trait grit (the perseverance and passion for long-term goals) had a small effect on performance, while working memory capacity had no impact. The authors concluded that time on task was the strongest predictor of performance in Dota 2.

However, as noted by Ding et al. [40], to their knowledge no studies have investigated plausible relationships between skills in MOBAs and decision-making ability. This is interesting, as decision-making constitutes an important aspect of many MOBA games. On a basic level, decision-making entails selecting between two or more options and is a common occurrence in our daily lives. According to the dual-process theory of judgment and decision making (e.g., [43,44]) humans rely on two “systems” or “processes”. One process is described as intuitive and fast, but is prone to errors. The other process is described as analytic and rule-based. Capitalizing on the dual-process theory, Séguin, Arseneault and Tremblay [45] also describe the ability to make decisions as being influenced by two different processes that operate during the different stages of decision-making (representing the problem, planning, executing and evaluating). One is a cognitive process, referred to as “cool” decision-making, using rational thinking and conscious consideration of different options and their risks and rewards. The other is an affective process, referred to as “hot” decision-making that is associated with emotional and visceral responses. It has been argued that learning in a decision-making task is dependent on regulation of affective processes and enabled by “cool” processes [45]. Bechara [46], on the other hand, argues that non-impaired decision-making is marked by subconsciously developing biases towards certain responses in uncertain tasks which guide us and are expressed through somatic responses or “gut” feelings, thus emphasizing the importance of visceral responses in decision-making. Of course, decision-making is an important aspect in everyday life, and there are differences between individuals when it comes to the ability to make good decisions, differences that coincide with individual differences in other cognitive functions (e.g., [47–49]). To be able to investigate factors related to differences in decision making in everyday life, it is therefore important to find measures that to some extent can mirror real life situations or settings.

A commonly used measure of decision-making is the Iowa Gambling Task (IGT), a card playing task designed by Bechara, Damasio, Damasio and Anderson [50]. The unique contribution of IGT is that it was developed to examine deficits in real-world decision-making, although in a laboratory setting. IGT has, for instance, been used to assess decision making such as in clinical conditions (see e.g., [51]), but also in non-clinical populations (see e.g., [52]) and in relation to action video gaming [53]. In the task, participants repeatedly make choices between four decks of cards. Once a card has been chosen, the player will win or lose an amount of money. Two of the four decks are ‘advantageous’, meaning that they produce small but constant gains over time and only include occasional losses. The two ‘disadvantageous’ decks produce large gains over time, but on the other hand, they also include even larger sporadic losses. To be successful in the IGT, the player needs to explore and, over time, learn the probabilities of risks involved. As noted, the IGT was developed to be similar to a real-life situation, and transfer to tasks with ecological validity are, of course, of extra interest in the area of cognitive training and transfer effects.

As noted, previous studies have not investigated how performance in so called MOBA games, in this study Dota 2, is related to decision making skills. In addition, there is only a sparse number of studies that have investigated how video game performance is related to decision making ability in tasks similar to real-life situations, as in the IGT. Although the challenges encountered in the IGT are not directly analogous to the ones in Dota 2, they both have in common the premise of decision-making under ambiguous circumstances, with few clear indicators of success. As both activities put demands on complex decision-making and higher-order cognitions it is possible that the specific skills needed when playing Dota 2 could be applied to the IGT, and possibly even to the decision-making present in everyday life.

Thus, this study aimed to examine the relationship between skill level in the video game Dota 2 and the ability to make decisions under ambiguity and experience as measured by the IGT. Such knowledge can give insight into the cognitive demands related to performance in Dota 2. Results may also give understanding about whether video gaming has the potential to cause (far) transfer effects to tasks other than those specifically trained, and if video games, Dota 2 in particular, can be useful as framework for the design of future interventions with regard to decision making ability. However, although it is plausible that long-term practice in Dota 2 may promote decision making ability, a reversed relationship cannot be excluded. This would suggest that decision making ability is a factor that rather makes individuals more likely to play Dota 2 and become successful. Thus, results from this study may also give some insight into whether decision making ability can be related to selection effects in Dota 2. In the analyses, we included a measure of matches played (time-on-task) in Dota 2, as well as a measure of conscious, analytical, rational, and logical thinking (CRT), factors that may either have a direct or an indirect influence on both performance in Dota 2 and decision-making ability.

## Method

### Participants

Participants were contacted through a post on a popular online forum for Dota 2 (https://www.reddit.com/r/DotA2/), a forum consisting of 607 000 members at the time of recruitment. A total of 557 Dota 2 players decided to participate in this study. However, after validation of data, and exclusion of participants who had not completed all tests and/or had performed repeated test sessions, the final sample consisted of 337 participants. Participants represented countries from all continents and among them there were 322 males, 3 females, 1 other, and 11 who preferred not to say. The mean age was 23.27 years (*SD* = 3.80) and mean years of education for the sample was 14.55 (*SD* = 3.65).

### Procedure

Requirements for participation were to be at least 16 years old, to have an updated match-making rating (MMR), and to have made match data public. The post explained the overall premises of the study and included a link to the study and the test material. Thus, all tasks and questionnaires were carried out online. On the site, the aim of the study was emphasized; that the study set out to examine the relationship between the ability to make decisions with lacking information and experience in Dota 2, because the complexity of the game makes it hard to know what the “right” thing to do is and therefore could be subject to training effects. A consent form was obtained before continuing to the test. Before starting the test, participants reported their email address, steam-ID (identifying their player account), ranking in the game (i.e. MMR) and number of matches played. The personal details gathered, e-mail and steam-ID, were used for gathering of objective data (see under material and methods) and to facilitate compensation for participating in the study. Since both Dota 2 and the cognitive tests were carried out in English, participants also had to rate their English proficiency on a scale from 0–10 (*M* = 8.91, *SD* = 1.26) to be able to establish that the participants had satisfactory language skills.

Compensation was given in the form of a “steam-key” giving access to games available at the Steam platform and offered to the first 200 participants. After the initial information was given, participants executed a decision-making task (the Iowa gambling task, see description below), and then they were redirected to a page on which they answered questions about demographic information and gaming history. Finally, participants performed a test of analytic and deliberative reasoning (Cognitive reflection task, see description below). Participation in this study took approximately 20 to 30 minutes.

### Measures

#### Match-making rating (MMR)

A player’s performance in Dota 2 is measured by their MMR. Winning a ranked game nets a gain of points between 25 to 30 points, while losing a ranked game nets a loss of points between -25 to -30. Thus, a higher MMR score is indicative of a higher skilled player [53]. A proprietary algorithm matches players of similar ranking to play with and against each other and the amount of rating points won or lost is determined by the disparity in rating between the different teams. The exact MMR score, which can be seen within the game, cannot be seen by others than the players themselves, and was therefore reported by the participants. In this sample, the mean MMR was 3857.52 (*SD* = 1286.42).

#### Medal

A player’s performance in Dota 2 is also illustrated in form of a medal. A player can make their match-making data public which enables third parties to get an objective measure of their ability. In this case, Dota 2 shows the ranking in the form of a medal, which is based on a span of MMR. Medals are ordered and represented by seven different titles (e.g., Guardian, Legend), and each medal/title is in turn represented by five stars (i.e., range; 1–35). Whereas MMR is self-reported, medal is an objective measure that was collected from Valve Corporation by using their steam-ID. The mean for Medal was 23.86 (*SD* = 7.34).

#### Matches played

Information on total number of matches played, sometimes denoted as time-on-task [42], can be found within the game and was self-reported by the participants. Sample mean for number of matches played was 4056.62 (*SD* = 2449.83).

#### Cognitive reflection test (CRT)

A 6-item version of the CRT task was used [54,55]. The test can be used as a measure of the tendency to engage in conscious, analytical, rational, and logical thinking rather than produce responses based on intuitions. Performance on the test has shown correlation with cognitive ability [56], as measured by subtests of the Wechsler Abbreviated Scale of Intelligence [57], and fluid intelligence [55], as measured by Set 1 of the Advanced Progressive Matrices [58]. Thus, the CRT is multidimensional, and was used here since it may capture many aspects relevant for performance in Dota 2 as well as in the Iowa gambling task. The CRT test included six open-ended problems/questions. For example, the first question reads as follows: “A bat and a ball cost £1.10 in total. The bat costs £1.00 more than the ball. How much does the ball cost?”. The answer can be one of three possible answer types: correct answer (£0.05), heuristic incorrect answer (£0.10) and non-heuristic incorrect answers (i.e. any other answer). The participants were also instructed to answer if they had seen that question before. The CRT was scored using the most typical approach of summing up all correct responses resulting in the CRT-Reflective Scoring, ranging from 0 to 6 [59]. Mean for CRT was 4.55 (*SD* = 1.54).

#### Iowa gambling task (IGT)

Decision-making skills were measured through the IGT. In this task, participants were instructed to pick between four buttons (A-D) each containing a gain of virtual money and sometimes also a loss. Participants were given a starting amount of 2000 and were told to maximize their winnings but not how many times they would have to click one of the buttons. Choosing buttons, A or B would gain the participant an amount of 100 for each click but would over the span of ten clicks incur a loss of 1250. Conversely, choosing buttons C or D would gain the participant an amount of 50 for each click but would over the span of ten clicks incur a loss of 250. Buttons A and C would on average incur a loss for every other click with button A’s losses spanning from 150 to 350 and button C’s losses spanning from 30 to 70. Buttons B and D would on average incur a loss every ten clicks with the loss being 1250 for B and 250 for button D. Therefore, decks A and B can be considered bad buttons since clicking on either of them will on average result in a Net Worth loss of 250 in the span of ten clicks. Decks C and D can be considered good buttons since clicking on those buttons will on average increase Net Worth by 250 in the span of ten clicks. These probabilities were not known to the participant in advance and the test ended after a hundred cards were drawn. The programming for the IGT was performed on PsyToolkit [60,61]. The probabilities and values were modified in line with the “four-deck format”, as described by Lin et al. [62], and the hint instruction removed to be in line with Fernie and Tunney’s [52] revised IGT.

Performance on the IGT was measured by Net Worth at the end of the task and by subtracting the number clicks on buttons A and B from the number of clicks on buttons C and D for each block of 20 clicks, resulting in 5 blocks of 20. This made it possible to examine the participants’ decision-making patterns and it follows the procedure of previous studies [50,63,64]. Cognitive testing took approximately 5 to 10 minutes and ended after the participants had made 100 clicks and their Net Worth at the end of the game was presented to them. Mean for the IGT was 2019.61 (*SD* = 1540.74).

### Statistical analyses

First, descriptive information was calculated on all variables included in the present study, and next bootstrapped correlations were performed on these variables. Descriptive information and correlation analyses (using bootstrapping) were calculated with IBM SPSS-26 [65]. In the path analyses, the direct effects of age, Medals/MMR, matches played, and performance in the cognitive reflection task (CRT) on decision-making performance in the IGT were investigated. Indirect effects of the relationship between matches played on IGT through Medals/MMR were also considered. Since Medals and MMR were highly correlated, their predictive power was investigated in separated models. Three fit indices were used to evaluate both models: the root-mean-square error of approximation (RMSEA), Bentler’s comparative fit index (CFI), and chi-square divided by the degrees of freedom (*χ2/df*). Both standardized and unstandardized parameter estimates were used to explore how each predictor variable was associated with performance in IGT. We used bootstrapped estimates of standard error, 95% confidence intervals, and *p*-values. The path analyses were conducted with IBM Amos SPSS-26 [66] using full information maximum likelihood (FIML) estimation.

## Results

Descriptive data of the study sample can be seen in Table 1.

**Table 1:** Descriptive statistics of the study sample (*n* = 337).

	Mean	SD	Skewness^a^	Kurtosis^b^
1. Age	23.27	3.80	0.47	0.74
2. MMR	3875.52	1286.42	0.05	-0.25
3. Medal	23.86	7.34	-0.39	-0.50
4. Matches Played	4056.62	2449.83	1.19	2.40
5. CRT	4.55	1.54	-1.10	0.50
6. IGT (net worth)	2019.61	1540.74	- 0.42	0.10

*Note*. MMR = Match-making rating, CRT = Cognitive reflection task, IGT = Iowa gambling task.

^a^Standard error = 1.33

^b^Standard error = 0.27.

For all variables, both skewness and kurtosis were indicative of normally distributed data. An upper limit of 7 for kurtosis, and 2 for skewness, have been suggested in the literature [67]. The results from the Bootstrapped correlations can be seen in Table 2.

**Table 2:** Bootstrapped correlations between variables included in the study.

	1	2	3	4	5	6
1. Age	-					
2. MMR	-.01	-				
3. Medal	-.04	**.93** **	-			
4. Matches played	.10	**.59** **	**.56** **	-		
5. CRT	.03	.11*	.10	.05	-	
6. IGT	.01	**.15** **	**.17** **	.10	**.16** **	-

*Note*. Number of bootstrap samples was 1000 in all cases. MMR = Match-making rating, CRT = Cognitive reflection task, IGT = Iowa gambling task.

*p < .05

**p < .01. Values in bold withstood Bonferroni adj. *p* < *α* = .05/15 = .0033.

As Table 2 shows, there were several significant correlations between variables, and most of them withstood control for multiple comparisons. As expected, there was a very high positive correlation between MMR and Medal, 95% BCa CI [.90, .96], as both are indicators of performance in Dota 2. There were also positive correlations for performance indicators MMR, 95% BCa CI [.52, .66], and Medal, 95% BCa CI [.49, .63], with matches played. Finally, MMR [.05, .25], Medal [.06, .27], and CRT [.05, .28] were all significantly related to performance in the IGT, and thus these factors may predict variations in IGT to a significant degree. Age was not significantly related to any factor included in the correlation analysis, therefore this factor was excluded from further analyses.

**Fig 1:**
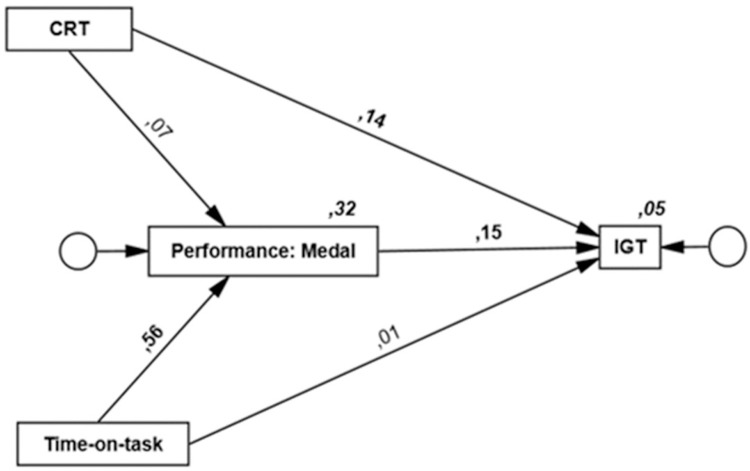
Path model with Iowa Gambling Task (IGT) regressed on Medal, cognitive reflection task (CRT), and Time-on-task. Digits on bold represent significant standardized regression weights. Digits in italics show squared multiple correlations.

Results from the model with Medal as the Dota 2 performance measure are shown in Fig 1 (standardized regression weights and squared multiple correlations) and Table 3 (unstandardized regression weights, 95% CI, standard error, and *p*-values).

**Table 3:** Regression weights in the model that included Medal, cognitive reflection task (CRT), and Time-on-task as predictors of performance in Iowa Gambling Task (IGT). Bootstrapped estimates were used to determine standard error, confidence intervals, and p-values.

Predictor variable		Outcome variable	B	LL (95% CI)	UL (95% CI)	S.E.	*P*
CRT	→	Medal	0.352	-0.052	0.823	0.234	.087
Time-on-Task	→	Medal	0.002	0.001	0.002	0.000	.010
CRT	→	IGT	144.662	17.240	249.164	55.122	.018
Time-on-Task	→	IGT	0.004	-0.070	0.090	0.039	.968
Medal	→	IGT	31.361	2.957	62.361	14.368	.020

*Note*: *B* = unstandardized regression weights. CI = Confidence Interval. LL = Lower Level. UL = Upper Level. *SE* = Standard Error.

Time-on-task was positively associated with Medal (Time-on-task → Medal, β = .56, *p* = .010) indicating that more time spent playing was associated with better performance on Dota 2. No association was observed between CRT and Medal. There was a positive association between Medal and IGT (Medal → IGT, β = .15, *p* = .019) suggesting that participants with higher score on Medal performed better on IGT. There was also a positive association between CRT and IGT performance (CRT → IGT, β = .14, *p* = .018). No association was found between Time-on-task and IGT. The only indirect effect found in the model suggested that Medal mediated a positive relationship between Time-On-Task and IGT performance (β_*ab*_ = .08, 95% CI [-.01, .16]). However, since there was no direct relationship between time-on-task and IGT even without indirect paths entered to the model (see also results from correlational analyses) it seems unlikely to suggest that Medal mediates any relationship between time spent playing Dota 2 and IGT performance (See e.g., [68]). The fit index for the Model was good, CFI = .1, RMSEA = .00, 90% CI [.00 –.14], and Chi-square (0.70)/*df* (1) = 0.70, *p* = .40.

**Fig 2:**
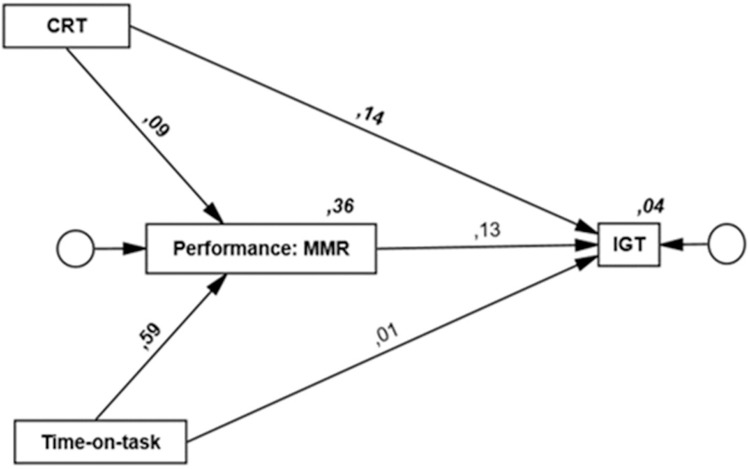
Path model with Iowa Gambling Task (IGT) regressed on match-making rating (MMR), cognitive reflection task (CRT), and Time-on-task. Digits on bold represent significant standardized regression weights. Digits in italics show squared multiple correlations.

Results from the second Model, using MMR as a measure of Dota 2 performance, are displayed in Fig 2 (standardized regression weights and squared multiple correlations) and Table 4 (unstandardized regression weights, 95% CI, standard error, and *p*-values).

**Table 4:** Regression weights in the model that included match-making rating (MMR), cognitive reflection task (CRT), and Time-on-task as predictors of performance in Iowa Gambling Task (IGT). Bootstrapped estimates were used to determine standard error, confidence intervals, and p-values.

Predictor variable		Outcome variable	B	LL (95% CI)	UL (95% CI)	S.E.	*P*
CRT	→	MMR	72.490	6.226	164.923	38.212	.044
Time-on-Task	→	MMR	0.309	0.256	0.381	0.031	.010
CRT	→	IGT	144.669	16.334	251.988	55.277	.018
Time-on-Task	→	IGT	0.009	-0.065	0.094	0.041	.845
MMR	→	IGT	0.152	-0.011	0.337	0.086	.068

*Note*: *B* = unstandardized regression weights. CI = Confidence Interval. LL = Lower Level. UL = Upper Level. *SE* = Standard Error.

Time-on-task was again positively associated with Dota 2 performance (Time-on-task → MMR, β = .59, *p* = .010) indicating that more time spent playing was associated with higher MMR. In this model, there was a positive association between CRT and performance on Dota 2 (CRT → MMR, β = .09, *p* = .044) signifying that better performance on CRT was related to higher MMR. There was no significant association between MMR and IGT (MMR → IGT, β = .13, *p* = .068). As in the previous model, there was a positive association between CRT and IGT performance (CRT → IGT, β = .14, *p* = .018) and no association was found between Time-on-task and IGT. No indirect effects were found for any of the variables in the model. The fit index for the Model was good, CFI = .1, RMSEA = .00, 90% CI [.00 –.14], and Chi-square (0.70)/*df* (1) = 0.70, *p* = .40.

Additional analyses were performed to test for non-linear relationships between variables included in the models. Curve estimation regression analyses revealed that in most cases linear relationships fitted the data best. However, with regards to players’ performance in Dota 2 (Medal, MMR), the analyses showed the strongest support for a S-curve relationship between Medal and IGT (*r*^2^ = .09, *p* = < .001), and between MMR and IGT (*r*^2^ = .08, *p* = < .001). This suggests a faster growth at the middle of the curve. That is, low levels of Dota 2 performance have limited influence on growth in IGT, medium level of Dota 2 proficiency is related to increased growth in IGT, followed by a slower rate of growth in IGT as players reach a higher level of proficiency in Dota 2.

## Discussion

The aim of the present study was to examine the relationship between skill level/performance in the video game Dota 2 and the ability to make decisions under ambiguity and experience as measured by performance in the Iowa gambling task (IGT). Results showed that Medal, an objective measure of ranking in Dota 2, was significantly predictive of performance in the IGT (Net Worth score). The other indicator used as measure of performance in Dota 2, the MMR score, revealed a borderline significant trend (*p* = 0.09) in relation to IGT. Additional analyses revealed that the relationship between performance in Dota 2 and IGT was particularly evident among participants that were at middle level range of Dota 2 proficiency (s-shaped curve relationship). The cognitive reflection task (CRT), used as measure of the ability to engage in conscious, analytical, rational, and logical thinking rather than to make decisions based on intuitions, was a significant predictor of performance in IGT as well, but also significantly related to MMR. However, there was no relationship between time-on-task (Matches Played in Dota 2) and IGT. As expected and based on previous findings [42,69], time-on-task proved to be a strong predictor of performance in Dota 2 (Medal, MMR).

Results from this study introduce the possibility that video gaming tapping into decision making under ambiguity can be used as a cognitive stimulating activity to promote decision making ability. More specifically, results from this study suggest that Dota 2 can potentially be used to increase decision-making ability. Although more studies are needed to confirm this finding, *if* video games can produce transfer into other areas of decision making, it may have great impact for future interventions on, for instance, elderly and clinical populations. It must be stressed though that the strength of the relationship between Medal and IGT was not very strong, and thus caution should be taken to avoid making overly strong claims of the findings. In addition, since the results from this study are based on cross-sectional findings (although measures from Dota 2 are based on time-consuming processes prior study baseline), it is not possible to determine the causal direction between factors. This raises the question of whether playing Dota 2 is a situation wherein the ability to make decisions can be developed, if those who are already better at decision-making play the game “better”, or both. If there is a “training effect” of playing Dota 2, it is possible that the challenges present in the game might help players develop more effective strategies for making conscious considerations and for regulating their affective processes (i.e. using rational thinking), as described by Séguin et al. [45]. It could also be that these challenges help players develop certain biases and make them more aware of their “gut-feelings”, as described by Bechara [46]. Findings from this study may thus, as noted above, indicate possible training effects that would suggest that performance in IGT could be linked to transfer from trained decision-making skills [18].

Results also showed that the relationship between level of performance in Dota 2 and IGT was not fully linear, and that there was faster growth in performance in IGT among participants who were in the middle range of Dota 2 proficiency. This may suggest that as one plays more Dota 2 and gets more skilled (passing a certain threshold), a more rapid growth in IGT performance will occur, but also that this relationship flattens out once you have reached a higher level of proficiency in Dota 2. Although speculative, from a cognitive training perspective, it is therefore possible that a certain minimum expertise level must be reached before transfer effects will be evident, and once one has reached high expertise (top level) the transfer effects will fade out. The possibility to improve results in IGT could be more difficult once one has achieved high scores and begins to approach maximum score.

If the opposite directionality is true, that better decision-makers are more likely to become skilled Dota 2 players, the results may suggest other possible implications. As e-sports are growing and becoming professionalized, identifying factors related to video game performance will probably become increasingly important. Thus, not only may the findings from this study help to understand the cognitive underpinnings related to performance in the game, it can potentially also be used as part of screening to identify individuals most likely to become successful in Dota 2. Regardless of the directionality between factors, results from this study highlights that related decision-making abilities are important aspects of “both games” (Dota 2 and IGT), which was also theorized in this study.

Notably, no direct effects of time-on-task (matches played) on IGT were found. This was somewhat surprising considering that practice obviously is strongly related to video game performance, and has been found to be a critical factor for development also in more traditional sports (see e.g., [70]). Results from analyses of indirect effects, which revealed a significant mediating effect (Time-on-task → Medal → IGT) should be interpreted with caution since no direct effect was found between time-on-task and IGT when no indirect relationships were included in the model [68]. This non-existing relationship between time-on-task and IGT was also confirmed in the correlational analyses. It could have been expected that the more one practices, the larger the improvements on decision making abilities, that in turn is related to performance in both Dota 2 and IGT. However, our findings suggest, as noted, that this is not true with regard to the relationship between time-on-task and IGT. It could still be the case that improved skills first has to be established (time-on-task → Dota 2 skill) before any transfer is present. Another possibility, of course, is that performance in IGT could reflect decision-making abilities that are predictive of success in Dota2, and thus less related to time-on-task, which may be rather influenced by other factors. As noted, the direction of causality between factors cannot be established in this study, and thus more experimental and longitudinal studies are needed to establish the directionality.

In this study, two indicators of performance in Dota 2 were used, namely Medal and MMR. One of them, Medal, was significantly related to IGT whereas the other, MMR, was not, although it should be noted that MMR was borderline significant. The reason it was less predictive may be because it was self-reported. The exact MMR score cannot be seen by others and was therefore reported by the participants. Medal, on the other hand, is an objective measure that was collected from Valve Corporation by using the players’ steam-ID. Although speculative, it may be that there was more noise in the MMR data than in Medal, which in turn caused this minor difference in predictive power. It must be stressed however that MMR scores have been used in previous studies (e.g., [42,69]) and have shown to be related to a number of other game related aspects (e.g., time-on-task).

To what degree Net Worth, used as dependent measure of IGT performance, is a measure of decision-making under ambiguity, on the one hand, and under risk, on the other, could be put to question. One could argue that the measure can be a kind of “composite” measure, containing or being dependent on both kinds of decision-making conditions. As such, what inferences can be made from this study? Firstly, results may suggest that different decision-making situations are present and important to handle also in Dota 2 (and perhaps in MOBAs as a whole), meaning that the game is demanding in different ways at once. Secondly, it seems that long-term decision-making (as measured with IGT) might be what benefits a player in the game. Interestingly, the current study also found that CRT, indicative of the ability of reflective thinking, was positively related to Dota 2 performance (MMR) as well as performance in IGT. CRT is also related to intelligence (e.g., [56]) and that intelligence may play a role for game expertise are in line with previous findings (e.g., [38]) but at odds with others. Röhlcke et al. [42], for instance, found no relationship between intelligence (Raven’s Matrices) and MMR score. However, the CRT is not a pure measure of general intelligence, it is rather a multidimensional measure and may thus capture a more comprehensive picture of what is important for Dota 2 as well as IGT performance than a “pure” measure of intelligence. It seems plausible that conscious, analytical, rational, and logical thinking, the dimensions of the CRT intended to capture reflective thinking, can be related to the performance measures used in this study. Thus, as it seems, a great strength of this study was the decision to include CRT to the analyses.

It has been questioned whether far transfer exists and that generalizability of such is hard to establish (e.g., [10,17]). *If* the results from this study are indicative of far transfer, and that video gaming can generate transfer to tasks not immediately related to those trained, it also must be stressed that the IGT actually was designed to distinguish between clinical and non- clinical populations (see e.g., [25,52,64]). Thus, it can be argued that this is a relatively “rough” measure. In this study, with a relatively homogenous sample with regard to age, gender, interest (gaming), and probably as well with regard to general health, we were still able to find observable differences on the IGT dependent on video gaming expertise. A question that remains is how results would have been on other measures of decision-making, and on tests developed to measure other aspects of decision-making than those included in this study. In addition, and as noted by Bediou et al. [21], different video games have different demands on our cognitive capacities. Thus, other MOBAs, or video games from other genres, could be even more (or less) predictive of IGT performance than skill in Dota 2. Future studies should therefore aim to differentiate the effects of different video games, and also include different outcome measures to be able to discriminate the potential effects of video gaming.

A great strength of this study was the use of a web-based approach, which allowed us to reach out to a large global community of young online video gamers. Another strength of this study was the large sample size, which increases the generalizability and reliability of the results. The study also included reliable cognitive tests such as the CRT and the IGT, which give further strength to our findings. The study also focused on a specific video game (Dota 2) in order to increase the possibility of being able to draw conclusions. As noted, it has been suggested that research should focus on the actual mechanics of the games played instead of using genre-based classifications [30].

However, in interpreting the results, some limitations should also be noted. Participants filled in questionnaires and performed all cognitive tests from a computer at home. Thus, we cannot rule out the possibility that instructions were not completely followed, or that participants rushed through the survey to acquire the compensation offered. Further, we were not able to control for the circumstances in which the respondents were tested, which can influence the results. However, it should be stressed that web-based studies often can be very reliable [71]. There are also some risks associated with self-reported measures (e.g., socially desirable answers), which in this study could possibly explain the slight difference in predictive power between Medal and MMR on IGT. To overcome the potential subjectivity induced form self-reported measures, we made efforts to also include objective measures.

The sample almost exclusively consisted of males. The number of females was so small that it was not justified to control for this factor. As a result, the study mainly examined the relationship between video gaming and decision-making in mostly males. Future studies investigating the influence of video gaming on cognitive abilities should therefore include games that also attract more females. In addition, the current study sample mostly included players of relatively high skill and experience. The sampling could have therefore masked, or enhanced, the effects of playing Dota 2. Future studies should thus aim to recruit a wider range of video gamers with regard to proficiency. Also, the results from this could be game-specific, and may thus not be generalizable to other games or genres.

Finally, it is always difficult to completely rule out the risk of participant reactivity. Although participants were informed about the overall aims of the study, and that playing video games potentially could be subject to training effects, in this study only skilled Dota 2 players were included, which potentially reduced the risk that participants altered their behavior differently according to “research expectations”. In addition, we did not give any specific information of what to expect from the cognitive tests. Thus, even if the players understood the overall aims of the study, and that they were expected to perform as well as possible, they did not know anything about expectations on *how* to execute the tests in a successful manner.

In conclusion, the present study suggests that skill level in Dota 2 is related to the ability to make decisions under ambiguity and experience as measured by the Iowa Gambling Task (IGT). This study presents novel evidence that playing strategic video games may contribute to increased decision-making abilities, which could suggest that playing video games can be used for cognitive interventions. However, it is also possible that decision-making abilities can to some extent predict performance in strategic video games, which should be of interest for the e-sport community when screening for players likely to be successful in e-sports. Future studies are however needed to replicate the findings from this study. Future studies would also benefit from a longitudinal design to establish the directionality between video gaming and decision-making ability.

## Supporting information

S1 Data (XLSX)

## References

[1] Wijman T. The Global Games Market Will Generate $152.1 Billion in 2019 as the U.S. Overtakes China as the Biggest Market. 2019. Available from: https://newzoo.com/insights/articles/the-global-games-market-will-generate-152-1-billion- in-2019-as-the-u-s-overtakes-china-as-the-biggest-market/ (Retrieved 2019-09-27).

[2] Dale G, Green CS. The Changing Face of Video Games and Video Gamers: Future Directions in the Scientific Study of Video Game Play and Cognitive Performance. Journal of Cognitive Enhancement. 2017; 1(3): 280–294. 10.1007/s41465-017-0015-6.

[3] Boyle EA, Hainey T, Connolly TM., Gray G, Earp J, Ott M, et al. An update to the systematic literature review of empirical evidence of the impacts and outcomes of computer games and serious games. Computers & Education. 2016; 94(C): 178–192. 10.1016/j.compedu.2015.11.003.

[4] Connolly TM, Boyle EA, MacArthur E, Hainey T, Boyle JM. A systematic literature review of empirical evidence on computer games and serious games. Computer & Education. 2011; 5(2): 661–686. 10.1016/j.compedu.2012.03.004.

[5] Nguyen L, Murphy K, Andrews G. (2019). Immediate and Long-Term Efficacy of Executive Functions Cognitive Training in Older Adults: A Systematic Review and Meta- Analysis. Psychological Bulletin. 2019; 145(7): 698–733. doi: 10.1037/bul0000196 30998045

[6] Karbach J, Verhaeghen P. Making Working Memory Work: A Meta-Analysis of Executive-Control and Working Memory Training in Older Adults. Psychological Science. 2014; 25(11): 2027–2037. doi: 10.1177/0956797614548725 25298292 PMC4381540

[7] Klingberg T, Fernell E, Olesen PJ, Johnson M, Gustafsson P, Dahlström K, et al. Computerized Training of Working Memory in Children With ADHD—A Randomized, Controlled Trial. Journal of the American Academy of Child & Adolescent Psychiatry. 2005; 44(2): 177–186. 10.1097/00004583-200502000-00010. 15689731

[8] Binder JC, Martin M, Zöllig J, Röcke C, Mérillat S, Eschen A, et al. Multi-Domain Training Enhances Attentional Control. Psychology and Aging. 2016; 31(4): 390–408. doi: 10.1037/pag0000081 27294719

[9] Zhao X, Xu Y, Huo X. The training of updating function: Content, effect and mechanism. Chinese Journal of Clinical Psychology. 2016; 24(5): 808–813.

[10] Melby-Lervåg M, Redick TS, Hulme C. Working Memory Training Does Not Improve Performance on Measures of Intelligence or Other Measures of “Far Transfer”: Evidence From a Meta-Analytic Review. Perspectives on Psychological Science. 2016; 11(4): 512–534. doi: 10.1177/1745691616635612 27474138 PMC4968033

[11] Thompson TW, Waskom ML, Garel KL, Cardenas-Iniguez C, Reynolds GO, Winter R, et al. Failure of working memory training to enhance cognition or intelligence. PLoS ONE. 2013; 8(5): e63614. doi: 10.1371/journal.pone.0063614 23717453 PMC3661602

[12] Ballesteros S, Mayas J, Prieto A, Eloisa R-M, Toril P, Reales JM. Effects of video game training on measures of selective attention and working memory in older adults: Results from a randomized controlled trial. Frontiers in Aging Neuroscience, 2017; 1(9): 354. doi: 10.3389/fnagi.2017.00354 29163136 PMC5671951

[13] Baniqued PL, Kranz MB, Voss MW, Lee H, Cosman JD, Severson J, et al. Corrigendum: Cognitive training with casual video games. Points to consider. Frontiers in Psychology. 2014; 5: 234. doi: 10.3389/fpsyg.2014.00234 24688477 PMC3960918

[14] Belchior P, Marsiske M, Sisco SM, Yam A, Bavelier D, Ball K, et al. Video game training to improve selective visual attention in older adults. Computers in Human Behavior. 2013; 29(4): 1318–1324. doi: 10.1016/j.chb.2013.01.034 24003265 PMC3758751

[15] Franceschini S, Gori S, Ruffino M, Viola S, Molteni M, Facioetti A. Action video games make dyslexic children read better. Current Biology. 2013; 23(6), 462–466. doi: 10.1016/j.cub.2013.01.044 23453956

[16] Toril P, Reales JM, Mayas J, Ballesteros S. Video game training enhances visuospatial working memory and episodic memory in older adults. Frontiers in Human Neuroscience. 2016; 10: 206. doi: 10.3389/fnhum.2016.00206 27199723 PMC4859063

[17] Sala G, Tatlidil KS, Gobet F. Video Game Training Does Not Enhance Cognitive Ability: A Comprehensive Meta-Analytic Investigation. Psychological Bulletin. 2018; 144(2): 111–139. doi: 10.1037/bul0000139 29239631

[18] Oei AC, Patterson MD. Playing a puzzle video game with changing requirements improves executive functions. Computers in Human Behavior. 2014; 37: 216–228. 10.1016/j.chb.2014.04.046.

[19] Large AM, Bediou B, Cekic S, Hart Y, Bavelier D, Green CS. Cognitive and Behavioral Correlates of Achievement in a Complex Multi-Player Video Game. 2019; 7(4): 198–212. 10.17645/mac.v7i4.2314.

[20] Bowman ND. Editorial: Video Games as Demanding Technologies. 2019; 7(4): 144–148. 10.17645/mac.v7i4.2684.

[21] Bediou B, Adams DM, Mayer RE, Tipton E, Green CS, Bavelier D. Meta-Analysis of Action Video Game Impact on Perceptual, Attentional, and Cognitive Skills. Psychological Bulletin. 2018; 144(1): 77–110. doi: 10.1037/bul0000130 29172564

[22] Roque NAA, Boot WRR. Action video games DO NOT promote visual attention. In Video Game Influences on Aggression, Cognition, and Attention (pp. 105–118). Springer International Publishing; 2018. 10.1007/978-3-319-95495-0_9.

[23] Wang P, Liu H, Zhu X, Meng T, Li H, Zuo X. Action Video Game Training for Healthy Adults: A Meta-Analytic Study. Frontiers in Psychology. 2016; 7: 907. doi: 10.3389/fpsyg.2016.00907 27378996 PMC4911405

[24] Bailey K, West R, Kuffel J. What would my avatar do? Gaming, pathology, and risky decision making. Frontiers in Psychology. 2013; 4: 609. doi: 10.3389/fpsyg.2013.00609 24058356 PMC3767905

[25] Buelow MT, Okdie BM., Cooper AB. The influence of video games on executive functions in college students. Computers in Human Behavior. 2015; 45(3): 228–234. 10.1016/j.chb.2014.12.029.

[26] Gan X, Yao Y, Liu H, Zong X, Cui R, Qiu N, et al. Action Real-Time Strategy Gaming Experience Related to increased Attentional Resources: An Attentional Blink Study. Frontiers in Human Neuroscience. 2020; 14:101. doi: 10.3389/fnhum.2020.00101 32341688 PMC7163005

[27] Gong D, Li Y, Yan Y, Yao Y, Gao Y, Yao D. The high-working load states induced by action real-time strategy gaming: An EEG power spectrum and network study. 2019; 131: 42–52. 10.1016/j.neuropsychologia.2019.05.002. 31100346

[28] Yao Y, Cui R, Li Y, Zeng L, Jiang J, Qiu N, et al. Action Real-Time Strategy Gaming Experience Related to Enhanced Capacity of Visual Working Memory. 2020; Frontiers in Human Neuroscience. 2020; 14: 333. doi: 10.3389/fnhum.2020.00333 33110407 PMC7489035

[29] Dale G, Kattner F, Bavelier D, Green CS. Cognitive abilities of action video game and role-playing video game players: Data from a massive open online course. Psychology of Popular Media. 2020; 9(3): 347–358. 10.1037/ppm0000237.

[30] Dale G, Green CS. Associations Between Avid Action and Real-Time Strategy Game Play and Cognitive Performance: a Pilot Study. Journal of Cognitive Enhancement. 2017; 1(6): 295–317. 10.1007/s41465-017-0021-8.

[31] Qiu N, Ma W, Fan X, Zhang Y, Li Y, Yan Y, et al. Rapid Improvement in Visual Selective Attention Related to Action Video Gaming Experience. Frontiers in Human Neuroscience. 2018; 12: 47. doi: 10.3389/fnhum.2018.00047 29487514 PMC5816940

[32] Dale G, Joessel A, Bavelier D, Green CS. A new look at the cognitive neuroscience of video game play. Annals of the New York Academy of Sciences. 2020; 1464. 10.1111/nyas.14295. 31943260

[33] Mora-Cantallops M, Sicilia M-Á. MOBA games: A literature review. Entertainment Computing. 2018; 26: 128–138. 10.1016/j.entcom.2018.02.005.

[34] Einhorn HJ, Hogarth RM. Ambiguity and Uncertainty in Probabilistic Inference. Psychological Review. 1985; 92(4): 433–461. 10.1037/0033-295X.92.4.433.

[35] Steamcharts. An ongoing analysis of Steam’s concurrent players; 2021. Available from: https://steamcharts.com/app/570 (Retrieved 2021-08-05).

[36] Statista. Number of monthly active users (MAU) of DOTA 2 worldwide; 2021. Available from: https://www.statista.com/statistics/607472/dota2-users-number/ (Retrieved 2021-08-05).

[37] E-sports earnings. Top Games Awarding Prize Money; 2021. Available from: https://www.esportsearnings.com/games (Retrieved 2021-08-05).

[38] Kokkinakis AV, Cowling PI, Drachen A, Wade AR. Exploring the relationship between video game expertise and fluid intelligence. PLoS ONE. 2017; 12(11): e0186621. doi: 10.1371/journal.pone.0186621 29141019 PMC5687598

[39] Wechsler D. Wechsler Abbreviated Scale of Intelligence–Second Edition (WASI-II). San Antonio, TX: NCS Pearson; 2011.

[40] Ding Y, Hu X, Li J, Ye J, Wang F, Zhang D. What Makes a Champion: The Behavioral and Neural Correlates of Expertise in Multiplayer Online Battle Arena Games. International Journal of Human-Computer Interaction. 2018; 34(8): 682–694. 10.1080/10447318.2018.1461761.

[41] Castaneda LK, Sidhu MKJ, Swanson T, Azose J. Game play differences by expertise level in Dota 2, a complex multiplayer video game. International Journal of Gaming and Computer-Mediated Simulations. 2016; 8(4): 1–24. 10.4018/IJGCMS.2016100101.

[42] Röhlcke S, Bäcklund C, Sörman DE, Jonsson B. Time on task matters most in video game expertise. PLoS ONE. 2018; 13(10): e0206555. doi: 10.1371/journal.pone.0206555 30372473 PMC6205640

[43] Evans JSBT Stanovich KE. Dual-process theories of higher cognition: advancing the debate. Perspectives on Psychological Science. 2013; 8(3): 223–241. doi: 10.1177/1745691612460685 26172965

[44] Kahneman D. Thinking, fast and slow. Farrar, Straus and Giroux, New York, NY; 2011.

[45] Séguin JR, Arseneault L, Tremblay RE. The contribution of "cool" and "hot" components of decision-making in adolescence: Implications for developmental psychopathology. Cognitive Development. 2007; 22(4): 530–543. 10.1016/j.cogdev.2007.08.006.

[46] Bechara A, Damasio AR, Damasio H, Anderson SW. Insensitivity to future consequences following damage to human prefrontal cortex. Cognition. 1994; 50(1–3): 7–15. doi: 10.1016/0010-0277(94)90018-3 8039375

[47] Del Missier F, Mäntylä T, Hansson P, Bruine de Bruin W, Parker AM, Nilsson L-G. The multifold relationship between memory and decision making: An individual-differences study. Journal of Experimental Psychology: Learning, Memory, and Cognition. 2013; 39(5): 1344–1364. doi: 10.1037/a0032379 23565790 PMC4160880

[48] Stanovich KE, West RF. Individual differences in reasoning: Implications for the rationality debate? Behavioral and Brain Sciences. 2000; 23(5): 645–665. doi: 10.1017/s0140525x00003435 11301544

[49] Stanovich KE, West RF. On the relative independence of thinking biases and cognitive ability. Journal of Personality and Social Psychology. 2008; 94(4): 672–695. doi: 10.1037/0022-3514.94.4.672 18361678

[50] Bechara A. The role of emotion in decision-making: Evidence from neurological patients with orbitofrontal damage. Brain and Cognition. 2004; 55(1): 30–40. doi: 10.1016/j.bandc.2003.04.001 15134841

[51] Buelow M, Suhr T. Construct Validity of the Iowa Gambling Task. Neuropsychology Review. 2009; 19(1): 102–114. doi: 10.1007/s11065-009-9083-4 19194801

[52] Fernie G, Tunney RJ. Some decks are better than others: The effect of reinforcer type and task instructions on learning in the Iowa Gambling Task. Brain and Cognition. 2006; 60(1): 94–102. doi: 10.1016/j.bandc.2005.09.011 16271818

[53] Dota 2 Gamepedia; 2021. Avaliable from https://dota2.gamepedia.com/Dota_2_Wiki (Retrieved 2021-08-05).

[54] Frederick S. Cognitive Reflection and Decision Making. Journal of Economic Perspectives. 2005; 19(4): 25–42. doi: 10.1257/089533005775196732

[55] Primi C, Morsanyi K, Chiesi F, Donati MA, Hamilton J. The Development and Testing of a New Version of the Cognitive Reflection Test Applying Item Response Theory (IRT). Journal of Behavioral Decision Making. 2016; 29(5): 453–469. 10.1002/bdm.1883.

[56] Toplak M, West E, Stanovich R. The Cognitive Reflection Test as a predictor of performance on heuristics-and-biases tasks. Memory & Cognition. 2011; 39(7): 1275–1289. doi: 10.3758/s13421-011-0104-1 21541821

[57] Wechsler D. Wechsler abbreviated scale of intelligence (WASI). San Antonio: Harcourt Brace, Psychological Corp; 1999.

[58] Raven JC. Advanced progressive matrices. London: Lewis & Co. Ltd; 1962.

[59] Pennycook G, Cheyne JA, Koehler DJ, Fugelsang JA. Is the cognitive reflection test a measure of both reflection and intuition? Behavior Research Methods. 2016; 48(1): 341–348. doi: 10.3758/s13428-015-0576-1 25740762

[60] Stoet G. PsyToolkit—A software package for programming psychological experiments using Linux. Behavior Research Methods. 2010; 42(4): 1096–1104. doi: 10.3758/BRM.42.4.1096 21139177

[61] Stoet G. (2017). PsyToolkit: A novel web-based method for running online questionnaires and reaction-time experiments. Teaching of Psychology. 2017; 44(1): 24–31. 10.1177/0098628316677643.

[62] Lin C-H, Chiu Y-C, Lee P-L, Hsieh J-C. (2007). Is deck B a disadvantageous deck in the Iowa Gambling Task? Behavioral and Brain Functions. 2007; 3(1): 16. 10.1186/1744-9081-3-16. 17362508 PMC1839101

[63] Fukui H, Murai T, Fukuyama H, Hayashi T, Hanakawa T. Functional activity related to risk anticipation during performance of the Iowa gambling task. Neuroimage, 2005; 24(1): 253–259. doi: 10.1016/j.neuroimage.2004.08.028 15588617

[64] Brand M, Kalbe E, Labudda K, Fujiwara E, Kessler J, Markowitsch HJ. Decision-making impairments in patients with pathological gambling. Psychiatry Research. 2005; 133(1): 91–99. doi: 10.1016/j.psychres.2004.10.003 15698681

[65] IBM Corp. IBM SPSS Statistics for Windows, Version 26.0. Armonk, NY: IBM Corp; 2019.

[66] Arbuckle JL. IBM SPSS AMOS 23 User’s Guide. Armonk, NY: IBM Corp; 2016.

[67] Finney SJ, DiStefano C. “Non-normal and categorical data in structural equation modeling,” in Structural Equation Modeling: A Second Course, eds Hancock G. R. and Mueller R. O. (Greenwich, Connecticut: Information Age Publishing), 269–314; 2006.

[68] Baron RM, Kenny DA. The moderator-mediator variable distinction in social psychological research: Conceptual, strategic, and statistical considerations. Journal of Personality and Social Psychology. 1986; 51: 1173–1182. doi: 10.1037//0022-3514.51.6.1173 3806354

[69] Hulaj R, Nyström MBT, Sörman DE, Bäcklund C, Röchkle S, Jonsson B. (2020). A Motivational Model Explaining Performance in Video Games. Frontiers in psychology. 2020; 11: 1510. doi: 10.3389/fpsyg.2020.01510 32760321 PMC7372929

[70] Baker J, Young BW. 20 years later: Deliberate practice and the development of expertise in sport. International Review of Sport & Exercise Psychology. 2014; 7(1): 135–157. 10.1080/1750984X.2014.896024.

[71] Gosling S, Vazire S, Srivastava S, John O. Should We Trust Web Based Studies? A Comparative Analysis of Six Preconceptions About Internet Questionnaires. American Psychologist. 2004; 59(2): 93–104. doi: 10.1037/0003-066X.59.2.93 14992636

